# Post-Cardiotomy Parasternal Nerve Block with Bupivacaine May Be Associated with Reduced Post-Operative Opioid Use in Children: A Retrospective Cohort Study

**DOI:** 10.3390/children7030020

**Published:** 2020-03-11

**Authors:** Francis X. Moga, Mark D. Lo Galbo, David M. Overman, Stefan J. Friedrichsdorf

**Affiliations:** 1 Children’s Heart Clinic PA, Minneapolis, MN 55404, USA; doverman@chc-pa.org; 2 Children’s Minnesota, Minneapolis, MN 55404, USA; 3 Mayo Clinic-Children’s Minnesota Cardiovascular Collaborative, Rochester/Minneapolis, MN 55905/55404, USA; 4 Children’s Research Institute, Children’s Minnesota, Minneapolis, MN 55404, USA; mark.logalbo@childrensmn.org; 5 Center of Pediatric Pain Medicine, Palliative Care and Integrative Medicine, Benioff Children’s Hospitals in Oakland and San Francisco, University of California at San Francisco UCSF, San Francisco, CA 94158, USA; 6 Department of Pain Medicine, Palliative Care and Integrative Medicine, Children’s Minnesota, Minneapolis, MN 55404, USA

**Keywords:** pediatric pain, congenital heart surgery, congenital heart disease, pain, postoperative care, parasternal nerve block, bupivacaine, opioid

## Abstract

Postoperative pain treatment affects immediate and long-term outcomes in children undergoing cardiac surgery. Opioids, as part of multimodal analgesia, are effective in treating pain, however, they can be disadvantageous due to adverse side effects. Therefore, we assessed whether the local anesthetic bupivacaine as a parasternal nerve block in children post-cardiac surgery is an effective adjunct to pain management. This was a retrospective cohort study of all patients who underwent cardiothoracic surgery via median sternotomy at a large children’s hospital between November 2011 and February 2014 with and without bupivacaine following the introduction of perioperative bupivacaine in late 2012 on a single unit. 62 out of 148 patients (age 3–17 years) who received bupivacaine demonstrated decreased postoperative opioid use. Within one day of surgery, patients who received bupivacaine required, on average, 0.57 mg/kg (95% CI, 0.46 to 0.68) of total morphine equivalent compared to 0.93 mg/kg (95% CI, 0.80 to 1.06) for patients who did not receive bupivacaine. This difference was statistically significant after adjusting for potential confounders (*p*-value = 0.002). Length of stay and intubation were shorter on average among patients who received bupivacaine, but these differences were not statistically significant after adjusting for potential confounders. The study results seem to suggest that the perioperative administration of bupivacaine may reduce opioid usage among children post-cardiotomy.

## 1. Introduction

Pain in hospitalized children remains common, under-assessed, and under-treated, with 24 to 80 percent of hospitalized pediatric patients experiencing moderate to severe pain [1,2,3,4,5,6,7,8]. Hospitalized children experiencing severe pain without adequate analgesia show negative long-term consequences, including a higher risk for post-traumatic stress disorder and increased morbidity and mortality [9,10,11,12,13]. Effective pain treatment after cardiac surgery in the pediatric population presents the challenge of balancing effective pain control with limiting opioid-induced side-effects, sedation, and respiratory effects [14,15,16,17,18,19,20].

An effective perioperative pain control regimen not only improves the quality of life of children undergoing open-heart surgery but facilitates the process of early extubation and assists with maintaining stable hemodynamics [14,21]. Following cardiac surgery, significant cerebral, physiological, and behavioral activity is present in response to a noxious procedure in critically ill children despite the administration of analgesic treatment [22]. Opioids, such as morphine, fentanyl, hydromorphone, oxycodone, and methadone (in the UK: Diamorphine), are often a key pillar of effective analgesia but might be associated with side effects including pruritus, urinary retention, nausea and vomiting, sedation, and respiratory depression [23,24,25].

Most advanced pediatric cardiac centers now appear to employ the concept of multimodal analgesia, i.e., combining modalities, which act synergistically for more effective (opioid-sparing) pediatric pain control with fewer side effects than a single analgesic or modality [24,26]. Additional mitigating strategies are not well explored in pediatric cardiac surgery and might include the administration of sodium-channel blockers, including perioperative lidocaine as a local anesthetic [27]. Bupivacaine, another local anesthetic agent and sodium-channel blocker most commonly used for epidural, spinal, and caudal anesthesia [28], has been considered as a useful adjunct to postoperative pain management strategies and been found safe to use in children [16]. Studies in adults have shown the use of bupivacaine—either direct application, infusion, or a combination—to reduce pain scores, opioid use, length of intubation, and length of hospital stay after cardiac surgery [29,30,31,32,33].

Two small studies (*n* = 34 children) reported improved analgesia as a result of continuous local infusion in a post-surgical pediatric population [34,35], but more evidence is warranted to establish the safety and efficacy of bupivacaine administered at the surgical site in vulnerable populations such as pediatric cardiac surgery patients. However, analgesic trials pose unique scientific, ethical, and practical challenges in pediatrics [36]. Berde et al. in their Food and Drug Administration (FDA) scientific report on pediatric analgesic clinical trial designs, measures, and extrapolation recommended using innovative study designs and outcome measures specific for children, including comparing the opioid use in the intervention and control group as a proxy for the efficacy of an analgesic modality [36].

As a result, this retrospective cohort study was undertaken to explore whether the perioperative administration of the local anesthetic bupivacaine as a parasternal nerve block might reduce opioid usage among children after cardiotomy on a pediatric cardiac intensive care unit.

## 2. Materials and Methods

The protocol for this retrospective cohort study was approved by the Institutional Review Board at Children’s Minnesota (IRB #1402-019, Date of Approval 02/25/14); informed consent was not required for a retrospective chart review. Following STROBE guidelines [37], the primary outcome of this observational study was to explore whether the perioperative administration of the local anesthetic bupivacaine as a parasternal nerve block might reduce opioid usage among children after cardiotomy on a pediatric cardiac intensive care unit. The secondary aim was to evaluate whether there would have been other clinical differences charted in the electronic medical records, such as pain scores, sedation scores, time to extubation, etc.

### 2.1. Setting

One of the largest freestanding children’s hospitals in the United States, with 429 staffed beds on 2 campuses and 5 intensive care units. The pediatric cardiac intensive care unit (Cardiovascular Care Center [CVCC]) has 25 beds and performs more than 400 open-heart surgeries annually.

### 2.2. Intervention

In addition to employing multimodal analgesia, triggered by clinical experience and emerging data [34], 2 cardiac surgeons (FXM, DMO) started in late 2012, administering the local anesthetic bupivacaine as a parasternal nerve block in the subject population. Within about 3 months, it was administered to all children expected to be extubated within 24 h. A quantity of 1.0 mL/kg of 0.25% bupivacaine solution without epinephrine was injected into the parasternal nerve bundles under direct vision at the end of the cardiac procedure. This local injection was placed under the intercostal nerves of 5–6 interspaces on each side of the sternotomy.

### 2.3. Patients

To assess potential clinical outcomes of this change of practice occurring in late 2012, patients prior and post-addition of bupivacaine were included. A chart review was conducted on pediatric patients undergoing cardiac surgery between 1 November 2011, and 1 February 2014. Eligible patients were between the ages of 3 and 17 years at the time of surgery, required a median sternotomy, and whose parents or caregivers consented to the general use of their medical information for research. Patients undergoing delayed sternal closure were excluded. Following surgery, all patients were admitted to the CVCC for postoperative care.

Specifically, there were 679 total cardiac cases during this time period, with 254 meeting the age requirement. The case total was verified by using the STS national database, which is an externally verified database. The chart review identified among those who were between 3 and 17 years of age 148 patients who had an initial operation at admission, a median sternotomy, and without ECMO support or further interventions. Those patients who met the study criteria had their information extracted from electronic medical records. Pre-exposure data included demographic information (e.g., gender, race), medical history (i.e., cardiac diagnosis, comorbidities, cardiac physiology), and description of the cardiac procedure (i.e., STAT [Society of Thoracic Surgeons-European Association for Cardio-Thoracic Surgery]—score mortality category, bypass time, aortic cross-clamp time, doses of cardioplegia). The exposure of interest was whether a patient received bupivacaine as a parasternal nerve block during surgery (bupivacaine group) or no bupivacaine (control group). Post-operative outcomes included length of intubation and hospital stay following surgery and the daily amount of medications administered.

Current pediatric pain trial recommendations include comparing opioid use in the intervention and control group as a proxy for the efficacy of an analgesic modality [36]. As such, this analysis focused on postoperative analgesic use on the day of and the day following surgery, because previous research with bupivacaine in adults has found that it significantly reduced postoperative pain for at least a 24-h period [29,32,33,38]. Specific analgesics of interest were opioids (i.e., fentanyl, hydromorphone, morphine) as well as basic analgesia (the non-steroidal anti-inflammatory ketorolac, and acetaminophen). The pharmacological aspect of the multimodal analgesia protocol on the CVCC during the study period included scheduled acetaminophen (initially intravenously, switched to enteral administration) and, if approved by the cardiac surgeon, intravenous ketorolac (switched to enteral ibuprofen or celecoxib). Regarding opioid use, patients most commonly received fentanyl, followed by hydromorphone, and/or morphine. The route of administration on the day of surgery and postoperative day 1 was a continuous opioid infusion plus a nurse- or patient-controlled analgesia (PCA) bolus. Starting doses (titrated to effect) were fentanyl 1 mcg/kg/h (max. 50 mcg, plus PCA bolus of 1 mcg/kg), hydromorphone 2–3 mcg/kg/h (max. 100–150 mcg, plus PCA bolus of 2–3 mcg/kg), or morphine 20 mcg/kg/h (max. plus PCA bolus of 20 mcg/kg). If opioid-induced side effects were not manageable by low-dose intravenous naloxone 0.5–2 mcg/kg/h, an opioid rotation was undertaken at equianalgesic doses [39]. The patients were transitioned to oral pain medications as soon as oral intake was safe and well-tolerated.

Daily measurements of pain, sedation, and withdrawal were also recorded (i.e., FLACC, SBS, WAT-1); however, they were excluded from this analysis because of inconsistent reporting into the electronic medical record system.

Based on pediatric opioid equivalent and conversion tables [24,39,40], the amount of intravenous morphine equivalent opioid use was computed and reported in milligram per kilogram body weight (mg/kg).

### 2.4. Statistical Analysis

The analysis was a comparison of the bupivacaine group to the control group. Pre-exposure characteristics were compared in order to assess potential confounding factors. Unadjusted analysis of the post-operative outcomes used the non-parametric Mann-Whitney test. Linear regression models were used to account for differences in patient characteristics between the 2 groups and incorporate important prognostic information. Outcome variables were transformed (i.e., log and square root) to satisfy standard regression assumptions, which were checked via diagnostic plots. Wald tests were used to test associations with the use of bupivacaine, adjusting for potential confounders. Both adjusted and unadjusted estimates of within-group averages were reported on the original scale. Adjusted estimates of transformed outcomes were back-transformed under standard regression assumptions; standard errors and confidence intervals were calculated via the bootstrap procedure.

Sensitivity analyses were performed. One compared different formulas for computing the amount of morphine equivalent (including adult opioid conversion tables) in order to ensure results were not dependent on the opioid used. Additional sensitivity analyses examined the impact of outliers. No specific subgroups or interactions were examined because an analysis of effect modification was beyond the scope of this study.

## 3. Results

### 3.1. Patient Characteristics

Of the 148 patients identified, 62 were administered bupivacaine during surgery, with the practice change starting in late 2012: In Nov-Dec 2011 *n* = 0/8 (0%) children received bupivacaine, in 2012 *n* = 11/88 (12.5%), in 2013 *n* = 46/57 (81%), and in January 2014 *n* = 5/5 (100%). The bupivacaine and control groups had similar ages, and the majority were white/non-Hispanic. The bupivacaine group had a higher percentage of females (*p* = 0.008, see Table 1). Both groups had typical BMI values for their age and sex and had a variety of cardiac diagnoses (see Table 2). Patients in the control group had more comorbidities than the bupivacaine group (*p* < 0.001, see Table 3). The two groups were similar with respect to intraoperative summaries. A majority of the procedures had STAT mortality category values of 1 or 2 (see Table 4). No changes to the anesthetic technique were noted during the time period of the study.

### 3.2. Opioid Use

**Figure 1:**
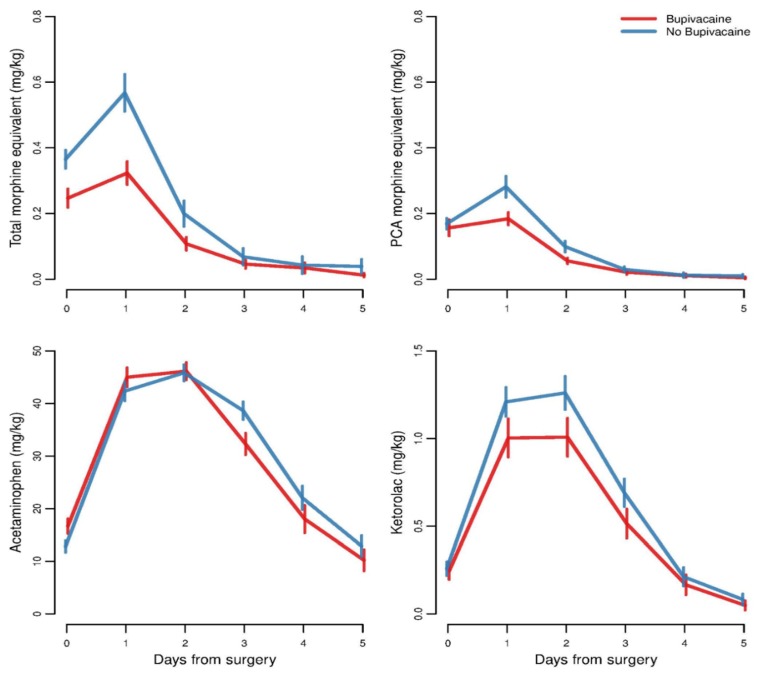
Change in the amount of pain medication administered for the first 5 postoperative days.

As expected, patients received the most morphine or opioid equivalent, both overall/total (continuous infusion of opioids and nurse- or patient-controlled analgesia (PCA) combined) and exclusively via nurse- or patient-controlled analgesia (PCA)-pumps, on the day of surgery and the following day, and usage dropped off quickly in subsequent days (see Figure 1). The largest difference in the amount of morphine equivalent used between the bupivacaine and control groups occurred during this initial spike in use. Over the course of the day of surgery and the following day, on average, the bupivacaine group used 0.57 mg/kg (95% confidence interval, 0.46 to 0.68) of total morphine equivalent compared to 0.93 mg/kg (95% confidence interval, 0.80 to 1.06) in the control group (see Table 5). On average, 0.34 mg/kg (95% confidence interval, 0.27 to 0.41) versus 0.45 mg/kg (95% confidence interval, 0.37 to 0.53) was PCA morphine equivalent use in the bupivacaine and control groups, respectively (see Table 5). This represented a 38% reduction of morphine equivalent use within one day of surgery, and these differences were statistically significant after adjusting for prognostic and potential confounding factors. As expected, the use of acetaminophen and ketorolac (since part of scheduled dosing protocol) were not associated with the use of bupivacaine (see Table 6). There was no significant difference in length of time in regards to PCA administration with the median length of PCA administration for both bupivacaine and control groups being 1.5 days (IQR for bupivacaine group was 0.93–1.84 while IQR for the control group was 0.95–2.26). Table 5 and Table 6 show the total morphine equivalent (all opioids administered by any route) and PCA morphine equivalent (opioids administered intravenously by PCA bolus only).

### 3.3. Time to Extubation

Patients in the bupivacaine group were intubated for 5.8 h (95% confidence interval, 4.0 to 7.5) following surgery compared to 8.9 h (95% confidence interval, 6.8 to 11.1) within the control group. This difference was not statistically significant after adjusting for confounding factors using linear regression models (see Table 6). Two patients who were intubated for over 100 h were excluded for analysis of intubation length. Both groups stayed in the hospital for an additional 6–7 days following surgery (see Table 6).

## 4. Discussion

An effective perioperative pain control regimen not only improves the quality of life of children undergoing open-heart surgery but also advances their clinical outcomes [14,21]. The less effective the postoperative pain treatment, the worse the immediate and long-term outcomes: A 10% increase in time spent in severe pain on postoperative day 1 resulted in a 30% increase of chronic post-surgical pain (CPSP) incidence at 12 months [41]. A recent systematic review and meta-analysis revealed a prevalence of CPSP in children across studies of 20% at 12 months after surgery [42]. Opioids continue to be the most effective analgesics and are frequently part of a postoperative pain protocol but might simultaneously be associated with significant dose-limiting side effects [23,24,25].

Multi-modal analgesia, i.e., combining modalities that act synergistically for more effective (opioid-sparing) pediatric pain control with fewer side effects than single analgesic or modality, is now the key approach in treating and preventing pain after cardiac surgery [24,26]. Multimodal analgesia post-cardiac surgery may include pharmacology such as (1) simple analgesia, e.g., acetaminophen, and non-steroidal anti-inflammatory drugs NSAIDs (or, if bleeding risk COX-2 inhibitor), (2) opioids, and (3) adjuvant analgesia, e.g., alpha-agonists [26] (dexmedetomidine, clonidine), gabapentinoids (gabapentin, pregabalin), NMDA-channel blockers (ketamine, the opioid methadone) may be combined with (4) regional anesthesia (e.g., neuroaxial infusion [epidural], peripheral/plexus nerve block, neurolytic block, intrathecal port/pump), (5) rehabilitation (e.g., physical therapy, graded motor imagery [43], occupational therapy), and, depending on the age of the child, (6) psychology (e.g., cognitive behavioral therapy), (7) spirituality (e.g., chaplain) and (8) integrative (“non-pharmacological”) modalities (e.g., mind-body techniques such as diaphragmatic breathing, bubble blowing, self-hypnosis, progressive muscle relaxation, biofeedback plus massage, aromatherapy, acupressure, acupuncture) [24,44,45].

This retrospective cohort study, exploring the practice change occurring on a large pediatric cardiac intensive care unit, seems to suggest that the perioperative administration of the local anesthetic bupivacaine as a parasternal nerve block may reduce opioid usage among children following cardiotomy.

There is limited evidence regarding the perioperative use of sodium-channel blockers in patients undergoing open-heart surgery. One recent study by Mattila et al. in 49 children aged 1–9 years could not demonstrate that a continuous ropivacaine wound infusion reduced morphine consumption, pain score or values, or nausea and vomiting in children who underwent atrium septum defect closure with median sternotomy and mediastinal drain [46]. One adult study on a continuous infusion of ropivacaine into the sternal wound following cardiac surgery was discontinued early due to an increased incidence of sternal wound infection [47]. In a pediatric randomized controlled study (RCT) with 72 children aged 3 months to 16 years undergoing a median sternotomy incision, a continuous incisional infusion of 0.25% levobupivacaine or bupivacaine reduced postoperative analgesic requirements and sedative use [35]. A small RCT in 30 children undergoing cardiac surgery with a median sternotomy receiving a 0.5% ropivacaine injection with 5 doses of 0.5 to 2.0 mL on each side in the 2nd to 6th parasternal intercostal spaces with a total dose of ropivacaine below 5 mg/kg or the same volume of saline before sternal wound closure resulted in lower opioid administration, lower pain scores, and earlier extubation [34]. Levobupivacaine and bupivacaine have rather similar pharmacological profiles [48], although research on bupivacaine alone in a pediatric setting is limited. Our cohort study adds to the body of pediatric literature that perioperative sodium-channel blockers, such as bupivacaine, are opioid-sparing postoperatively.

## 5. Study Limitations

Limitations of this study stem from collecting observational data from a retrospective open-enrollment cohort. A clinical practice change occurred in late 2012 with the addition of perioperative bupivacaine administration, and the majority of the control group consists of children prior to this point in time, as well as a few children who were not expected to be extubated within 24 h. As such, there are differences in terms of comorbidities and gender. An adjusted analysis accounting for these observed differences and other prognostic factors attempted to control for these differences. However, there may be other potential confounding factors that were not measured, and that might explain the observed associations. We were unable to account for daily measurements of pain and sedation via commonly used instruments (e.g., FLACC), due to inconsistent reporting. Clinicians in this study charged with managing patient pain post-surgery were not blinded to the use of a parasternal nerve block. This information may have influenced their decision to provide certain types and amounts of analgesics. However, the opioid analgesia was patient- or nurse-administered, and it appears less likely that the knowledge of a perioperative block may have influenced analgesia administration.

## 6. Conclusions

Despite the use of multi-modal analgesia, adequate pain treatment and prevention after pediatric cardiac surgery continues to represent the challenge of balancing effective pain control with limiting opioid-induced side-effects, sedation, and respiratory effects [14,15,16,17,18,19,20]. Additional evidence regarding analgesic modalities to improve immediate postoperative and long-term outcomes is needed. Our findings add to the body of literature and seem to suggest that bupivacaine for a parasternal nerve block in pediatric patients following cardiac surgery might be an advantageous adjunct in the post-surgical multi-modal pain management. Prospective, double-blinded pediatric RCTs would be required to support or refute the results of this retrospective open-label cohort study.

## Figures and Tables

**Table 1:** Patient demographic information.

	Bupivacaine (*n* = 62) *n* (%) *	Control (*n* = 86) *n* (%) *	*p*-Value
**Age (Year)**			
Mean (SD)	8.3 (4.5)	9.1 (4.7)	0.288
**Sex**			
Female	41 (66)	45 (52)	0.008 ^a^
**Race**			
Non-Hispanic White	45 (73)	59 (69)	0.762
Black	7 (11)	9 (10)	
Other, unknown or declined	10 (16)	18 (21)	
**BMI**			
Age and sex adjusted z- mean (SD)	0.1 (1.5)	−0.2 (1.4)	0.217
**Year of surgery**			
2011–2012	11 (18)	75 (87)	<0.001 ^a^
2013–2014	51 (82)	11 (13)	

* Unless indicated otherwise. ^a^ Significant difference between the two groups at α = 0.05 level SD: Standard deviation.

**Table 2:** Patient clinical information (cardiac surgical diagnosis).

	Bupivacaine (*n* = 62) *n* (%) *	Control (*n* = 86) *n* (%) *	*p*-Value
Pulmonary valve repair/replacement **	20 (32)	35 (41)	*N*/A
Mitral valve repair/replacement **	8 (13)	3 (3)	
Tricuspid valve repair/replacement **	5 (8)	13 (15)	
Aortic valve repair/replacement **	8 (13)	21 (24)	
Atrial septal defect repair/closure **	13 (21)	13 (15)	
Ventricular septal defect repair/closure **	3 (5)	10 (12)	
Patent foramen ovale repair/closure **	9 (15)	15 (17)	
Anomalous coronary artery repair **	4 (6)	6 (7)	
Fontan procedure **	10 (16)	7 (8)	
Pulmonary artery procedure **	9 (15)	14 (16)	
Right ventricular muscle bundle resection **	6 (10)	8 (9)	
Pacemaker procedure **	4 (6)	5 (6)	
Other procedure **	15 (24)	22 (26)	
**Fontan Physiology**			
Yes	11 (18)	8 (9)	0.206

* Unless indicated otherwise. ** Patients can have multiple interventions.

**Table 3:** Patient clinical information (comorbidities).

	Bupivacaine (*n* = 62) *n* (%) *	Control (*n* = 86) *n* (%) *	*p*-Value
None	42 (68)	33 (38)	<0.001 ^a^
Genetic- any **	6 (10)	18 (21)	0.075
Genetic- type			
Down Syndrome	3 (5)	5 (6)	
DiGeorge Syndrome	1 (2)	3 (3)	
Noonan Syndrome	0 (0)	1 (1)	
Marfan Syndrome	1 (2)	0 (0)	
Other	1 (2)	9 (10)	
Respiratory **	9 (15)	12 (14)	1
Neurological, developmental or psychological **	7 (11)	24 (28)	0.015 ^a^
Endocrine **	1 (2)	2 (2)	1
Musculoskeletal **	2 (3)	4 (5)	1
Renal or Urinary **	1 (2)	4 (5)	0.4
Gastrointestinal **	1 (2)	5 (6)	0.401
Hematologic/Lymphatic **	1 (2)	8 (9)	0.08
Other **	2 (3)	10 (12)	0.075

* Unless indicated otherwise. ** Patients can have multiple comorbidities. ^a^ Significant difference between the two groups at α = 0.05 level.

**Table 4:** Patient operative information.

	Bupivacaine (*n* = 62) *n* (%) *	Control (*n* = 86) *n* (%) *	*p*-Value
**STAT Category**			0.569
1	20 (32)	22 (26)	
2	28 (45)	37 (43)	
3	12 (19)	24 (28)	
4	1 (2)	3 (3)	
Missing	1 (2)	0 (0)	
**Aortic Cross Clamp Time**			0.317
Minutes- mean (SD)	49 (37)	55 (36)	
Missing	12 (19)	8 (9)	
**Bypass Time**			0.422
Minutes- mean (SD)	70 (39)	82 (52)	
Missing	1 (2)	1 (1)	
**Cardioplegia**			0.090
Doses- mean (SD)	1.5 (0.8)	1.8 (1.0)	
Missing	12 (19)	8 (9)	

* Unless indicated otherwise SD: Standard deviation STAT: Society of Thoracic Surgeons—European Association for Cardio-Thoracic Surgery.

**Table 5:** Outcome characteristics (unadjusted analysis).

	Bupivacaine (*n* = 62)	Control (*n* = 86)	Difference	*p*-Value
Total Morphine Equivalent, days 0–1 (mg/kg)	0.57 (0.46, 0.68)	0.93 (0.80, 1.06)	−0.36 (−0.53, 0.19)	<0.001 ^a^
PCA Morphine Equivalent, days 0–1 (mg/kg)	0.34 (0.27, 0.41)	0.45 (0.37, 0.53)	−0.11 (−0.21, 0.01)	0.049 ^a^
Acetaminophen, days 0–1 (mg/kg)	62 (56, 67)	55 (50, 60)	7 (−1, 14)	0.120
Ketorolac, days 0–1 (mg/kg)	1.2 (1.0, 1.5)	1.5 (1.2, 1.7)	−0.2 (−0.6, 0.1)	0.235
Length of intubation (hours) *	5.8 (4.0, 7.5)	8.9 (6.8, 11.1)	−3.1 (−5.9, −0.4)	0.004 ^a^
Length of hospital stay, post-surgery (days)	6.8 (5.6, 7.9)	7.1 (5.7, 8.5)	−0.3 (−2.1, 1.5)	0.775

Data presented as estimated mean (95% confidence interval). * 2 control patients were excluded because intubation length > 100 h. ^a^ Significant difference between the two groups at α = 0.05 level. PCA: Patient-Controlled Analgesia.

**Table 6:** Outcome characteristics (adjusted analysis).

	Bupivacaine (*n* = 61)	Control (*n* = 86)	Difference	*p*-Value
Total Morphine Equivalent, days 0–1 (mg/kg)	0.57 (0.46, 0.69)	0.92 (0.78, 1.05)	−0.34 (−0.54, −0.14)	0.002 ^a^
PCA Morphine Equivalent, days 0–1 (mg/kg)	0.30 (0.24, 0.36)	0.48 (0.40, 0.56)	−0.18 (−0.30, 0.07)	0.005 ^a^
Acetaminophen, days 0–1 (mg/kg)	56 (50, 63)	59 (54, 64)	−3 (−12, 7)	0.607
Ketorolac, days 0–1 (mg/kg)	1.5 (1.2, 1.8)	1.3 (1.0, 1.5)	0.2 (−0.2, 0.6)	0.442
Length of intubation (hours) *	5.6 (4.3, 6.9)	7.8 (6.0, 9.6)	−2.2 (−4.6, 0.2)	0.069
Length of hospital stay, post-surgery (days)	6.1 (5.3, 6.9)	6.9 (5.9, 7.8)	−0.7 (−2.0, 0.6)	0.291

Data presented as estimated mean (95% confidence interval). * 2 control patients were excluded because intubation length > 100 h. ^a^ Significant difference between the two groups at α = 0.05 level. PCA: Patient-Controlled Analgesia. Please note: For all of these outcomes the linear regression models adjusted for age, age squared, race (white, not white), comorbidities (genetic, respiratory, neurological, and other), STAT category (1, 2, 3/4), and year of surgery (2011/2012, 2013/2014) as potential confounders; 1 patient was excluded due to a missing STAT category. For the total morphine equivalent and PCA morphine equivalent outcomes, the analysis was performed after square root transformation. For the length of intubation and hospital stay outcomes, the analysis was performed after log transformation.
